# Impact of Surface Treatment on the Functional Properties Stainless Steel for Biomedical Applications

**DOI:** 10.3390/ma13214767

**Published:** 2020-10-26

**Authors:** Marcin Basiaga, Witold Walke, Magdalena Antonowicz, Wojciech Kajzer, Janusz Szewczenko, Alina Domanowska, Anna Michalewicz, Marek Szindler, Marcin Staszuk, Miłosz Czajkowski

**Affiliations:** 1 Faculty of Biomedical Engineering, Silesian University of Technology, Roosevelta 40, 41-800 Zabrze, Poland; witold.walke@polsl.pl (W.W.); magdalena.antonowicz@polsl.pl (M.A.); wojciech.kajzer@polsl.pl (W.K.); janusz.szewczenko@polsl.pl (J.S.); 2 Institute of Physics—Centre of Science and Education, Silesian University of Technology, 44-100 Gliwice, Poland; alina.domanowska@polsl.pl (A.D.); anna.michalewicz@polsl.pl (A.M.); 3 Faculty of Mechanical Engineering, Silesian University of Technology, Konarskiego 18A, 44-100 Gliwice, Poland; marek.szindler@polsl.pl (M.S.); marcin.staszuk@polsl.pl (M.S.); 4 Technolutions Sp.zo.o., Otolice 38, 99-400 Łowicz, Poland; milosz@technolutions.pl

**Keywords:** ZnO layer, ALD method, 316LVM steel, electropolised, sandblasted

## Abstract

The main goal of the carried out tests was to analyze the influence of the surface modification of a substrate by depositing composite ZnO layers by the Atomic Layer Deposition (ALD) method. The samples were subjected to preliminary surface modification consisting of being sandblasted and electropolished. A ZnO layer was applied to the prepared substrates by the ALD method. As a precursor of ZnO, diethylzinc (DEZ) was used, which reacted with water, enabling the deposition of the thin films. The chamber temperature was as follows: T = 100–300 °C. The number of cycles was 500 and 1500. As part of the assessment of the physicochemical properties of the resulting surface layers, the tests of chemical composition of the layer, pitting corrosion, impedance corrosion, adhesion to the metal substrate, morphology surface, and wettability were carried out. On the basis of the obtained research, it was found that a composite ZnO layer deposited onto a substrate previously subjected to the electrochemical polishing process has more favorable physicochemical properties. Moreover, an influence of temperature and the number of cycles of the deposition process on the obtained properties was observed, where the ZnO layer was characterized by more favorable properties at a temperature of 200–300 °C at 1500 cycles of the deposition process.

## 1. Introduction

Interference with the biological environment of the human organism disturbs its existing state of balance, and thus triggers defense reactions aimed at restoring full physiological efficiency. Bearing this in mind, it seems obvious to care for such physicochemical properties of implants that will cause the least undesirable reactions of rejection of the implanted “foreign matter”. The overall success of the method of treatment of a specific pathology is therefore influenced by a number of closely related factors. In addition to the treatment technique, the knowledge and experience of the medical personnel and the pharmacotherapy, the functional features obtained at the production stage of the medical device are also important. They consist of, among other things: the correct formation of the structure of the biomaterial, its mechanical properties as well as the physicochemical properties of the surface. In addition, the specific nature of the work environment of a medical device should be taken into account as well as the technique of implantation. The surface of the medical device, which in this case should be biocompatible, is responsible for the proper bond between the implant and the biological environment. The combination of the advantages of metal implant structures with the biocompatibility required for medical applications is achieved through various methods of the surface treatment of implants such as mechanical and electrochemical polishing, sandblasting, chemical passivation, production of oxide coatings obtained by the sol-gel method [1,2], chemical vapor deposition [3], plasma enhanced chemical vapor deposition [4], and atomic layer deposition [5,6,7]. A very important issue in the case of surface modification, in addition to the production of a biocompatible layer, is also the appropriate preparation of the metallic substrate. Suitable surface morphology should ensure, among other things, very good adhesion to the surface of the substrate. Therefore, the authors of this work proposed modification of the 316LVM steel surface through electrochemical polishing and sandblasting. The selection of the proposed modification of the 316LVM steel substrate was dictated by the requirement to obtain different values of surface roughness. This is very important because the proposed technology of atomic layer deposition (ALD) surface modification inherits the shape of the substrate surface, which may translate into its later use. As is well-known, implants with a smooth surface isolate the settling of bacteria on them, while a more developed surface results in faster osseointegration. Therefore, the properly prepared surface of the metallic substrate is very important. At present, it is believed that 60% to 80% of infections encountered in the body after implantation are related to the formation of biofilms. Biofilm-related infections are usually chronic, often life-threatening, and biofilm-forming bacteria are especially resistant to antibiotics and human immune mechanisms. Reducing the risk of biofilm development on the surface of biomaterials is one of the primary goals of researchers looking for effective strategies to prevent biofilm-related infections. The main approaches involve modification of the surface of the materials to reduce microbial adhesion and interference in the initial phase of biofilm development. In the literature, one can find a number of data related to the application of antibacterial layers. The most commonly used include oxides of silver, copper, and zinc [8,9,10,11]. However, the literature data indicate a number of undefined phenomena accompanying the production of antibacterial layers [12,13,14,15,16,17,18] on the surfaces of metal materials. The still unresolved problem is the selection of appropriate parameters of the production of the layers and appropriate preparation of the substrate as well as the lack of comprehensive studies showing the full characteristics of their behavior under implantation conditions and long-term contact with the tissue environment during the use of the implant. Therefore, the authors of the study attempted to analyze the influence of the surface modification of a substrate by depositing composite ZnO layers by the ALD method under specific technological conditions. The surfaces of the 316 LVM steel were prepared in two ways: sandblasted and polished.

## 2. Materials and Methods

Stainless steel (316LVM) samples obtained from a bar of diameter d = 14 mm were used in the studies. The samples were subjected to preliminary surface modification consisting of sandblasting and electropolishing. The sandblasting process was carried out using glass balls with diameters ranging from 50 to 150 μm on a Micra 2 precision sandblaster from Dentalfarm (Bielsko-Biała, Poland). The applied time was equal to t = 2 min and the pressure was p = 3 bar. Electrochemical polishing was performed by using a solution of phosphate-sulfate. A ZnO layer was applied to the prepared substrates by the ALD method. Zinc oxide thin films were deposited by the atomic layer deposition method using a R-200 standard system from Picosun (Helsinki, Finland). As a precursor of zinc oxide, diethylzinc was used, which reacted with deionized water, enabling the deposition of the thin films. The chemical reactions in the atomic layer deposition process were thermally assisted. The layers were deposited in a reaction chamber, also called a growth chamber, into which precursors were inserted cyclically, which can be in the solid, liquid, or gaseous phase. The chamber temperature was as follows: 100 °C, 200 °C, and 300 °C. The research was carried out for two different number of cycles: 500 and 1500. The dosing time for the reagents was 0.1 s. Nitrogen was used as a transporting gas and to purge the chamber between dosing cycles. Its flow was 200 SCCM and the flushing time was 4 s.

### 2.1. Chemical Composition of the Layer

The chemical composition of the passivation layer was studied by Auger electron spectroscopy combined with ion sputtering. The experiment was performed using a scanning Auger nanoprobe system manufactured by Physical Electronics, model PHI-670. The device was equipped with a Schottky field emission electron source and a full cylindrical mirror analyzer with a coaxial multichannel detector. The primary electron beam energy was 10 keV and 20 nA of the sample current, and was directed to the examined surface at an angle of 30°. In-depth profiling was carried out using an ion gun with the ion energy set to 1 keV to minimize atomic mixing on the interfaces and chemical effects induced by the ion beam. The angle of the ion beam and the normal of the sample was set at 58.6°. The spectra were averaged from the area of 60 × 80 µm size, while the whole sputtered crater size was set at 1 mm^2^. To achieve a high depth resolution, the alternating mode of the 1 min sputtering and spectra measurement was applied. The pressure in the main chamber while measuring the Auger electron spectra was maintained at 10^−7^ Pa.

### 2.2. Potentiodynamic Test

The resistance to pitting corrosion was tested by the potentiodynamic method, meeting the requirements of the ASTM F2129 standard [19], with the use of a Voltalab PGP201 potentiostat (Radiometer, Villeurbanne Cedex, Lyon, France). The reference electrode was that of the KP-113 saturated calomel electrode (SCE), while the auxiliary one was a PtP-201 platinum electrode. Corrosion tests were started by determining the Eocp open circuit potential in currentless conditions, and then polarization curves were recorded. Polarization curves were recorded from the value of the initial potential Eini = Eocp − 100 mV. The scan rate was equal to 0.167 mV/s. On the basis of the obtained curves, the breakdown potential Eb and using the Tafel method, the corrosion potential Ecorr and the value of polarization resistance Rp were determined. Additionally, the corrosion current icorr and the βa and βc coefficients were determined [6].

### 2.3. Impedance Test

In addition, studies were conducted using electrochemical impedance spectroscopy (EIS). The measurements were carried out with the use of the AutoLab PGSTAT 302N measurement system equipped with the FRA2 module (frequency response analysis). The same electrode system was used in the tests as in the chronoamperometric tests. The tested system’s impedance spectra are presented in the form of Nyquist diagrams for various frequency values (10^4^–10^−3^ Hz) and the form of Bode diagrams. The amplitude of the sinusoidal voltage of the excitation signal was 10 mV. The spectra obtained were interpreted after fitting the least squares method to the equivalent circuit. On this basis, numerical values of resistance R and capacitance C of the analyzed systems were determined [6,20,21].

Both the potentiodynamic and impedance tests were carried out in Ringer’s solution (NaCl—8.6 g/L, KCl—0.3 g/L, CaCl_2_⋅6H_2_O—0.48 g/L) (250 mL), supplied by Baxter, at T = 37 ± 1 °C and pH = 7 ± 0.2.

### 2.4. Scratch Test

The adhesion test was carried out using the scratch test method according to the standard [22]. The test was carried out at a force of up to 30 N with the following parameters: loading speed 10 N/min, speed of the table displacement 10 mm/min, and length of the scratch ~3 mm [7]. The tests of adhesion to the substrate were assessed based on microscopic observations and the friction force.

### 2.5. Scanning Electron Microscope (SEM)

The surface morphology of the prepared samples was evaluated with the use of a SUPRA 25 scanning electron microscope by ZEISS (Germany), within a magnification range of 1000–100,000×.

### 2.6. Wettability

In order to determine the surface wettability of the selected samples, the wetting angle and surface free energy (SFE) were evaluated with the use of the Owens–Wendt method. The wettability angle measurement were performed with two liquids: distilled water and diiodomethane. Measurements with a drop of liquid and diiodo-methane spread over the sample surface were carried out at room temperature at the test stand incorporating a Surftens Universal goniometer by OEG (Germany), and a PC, with Surftens 4.5 software to assess the recorded drop image [6].

## 3. Results

### 3.1. Chemical Composition of the Layer

**Figure 1:**
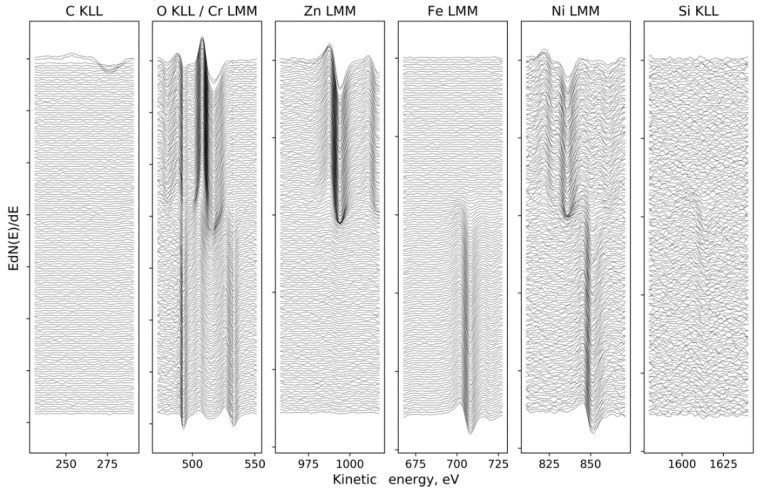
Sandblasted 1500_300: the evolution of the spectral lines for the major element, registered between ion etching cycles, ordered from surface (top) to the substrate (bottom).

**Figure 2:**
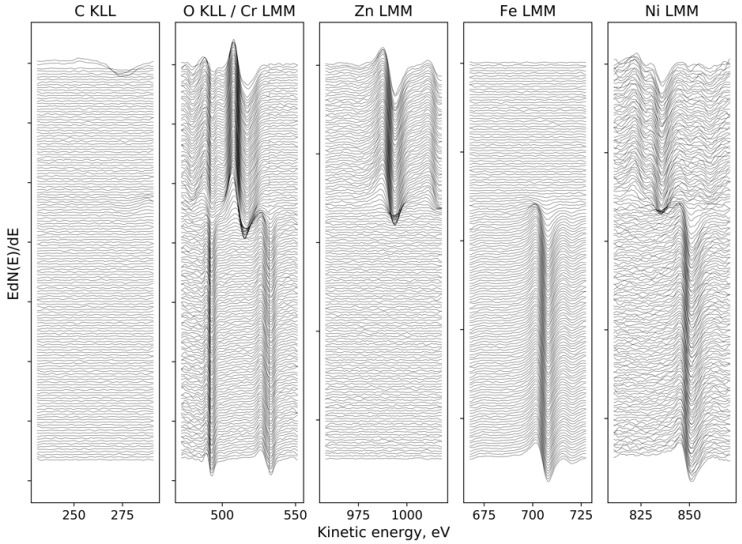
Electropolished 1500_300: the evolution of the spectral lines for the major element, registered between ion etching cycles, ordered from surface (top) to the substrate (bottom).

**Figure 3:**
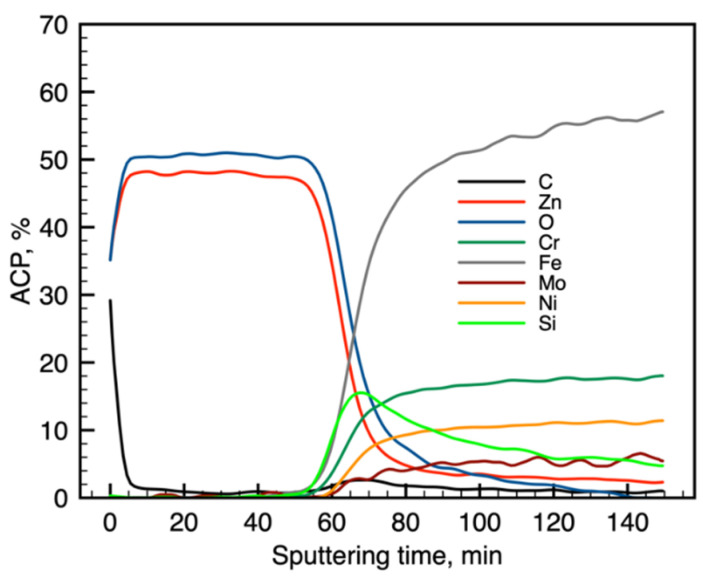
Sandblasted 1500_300: calculated atomic concentration in the depth profile.

**Figure 4:**
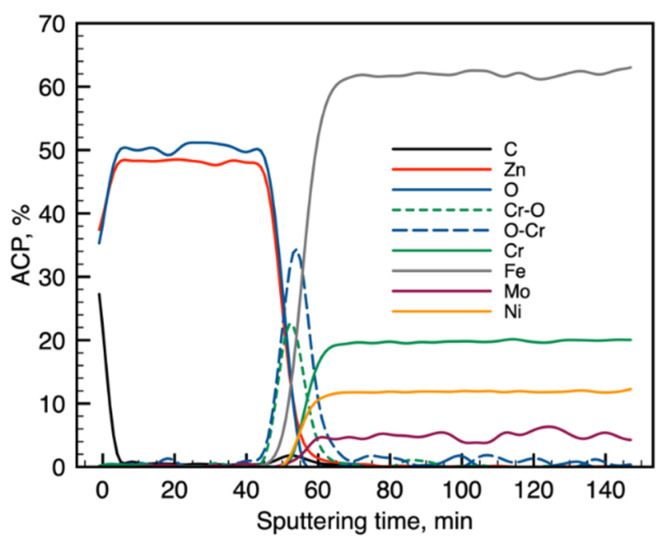
Electropolished 1500_300: calculated atomic concentration in the depth profile.

Figure 1 and Figure 2 present the Auger electron differentiated spectra, divided into energetic areas for the main elements of the structure of the Electropolished 1500_300 (O KLL and Cr LMM series presented on a common graph, because of the energy range overlapping; Zn LMM, Ni LMM, and Fe LMM series, and additionally C KLL), ordered in stacks, to illustrate the important chemical changes taking place with the depth. The top spectrum on each stack originates from the surface of the sample, while the consecutive spectra come from the increasingly deep areas of the structure being revealed by the ion sputtering. The evolution of the spectral lines versus sputtering cycles in terms of variations of their shape and energy position indicate changes in chemical composition with the sample depth. The spectra originating from the elements forming the oxide passivation layer do not change their shape or position with the depth, which indicates chemical homogeneity of the ZnO layer. It is evident that a significant change occurs at the substrate–passivation layer interface. It should be highlighted that the main advantage of the AES technique dedicated for surface analysis is that Auger electrons are emitted mainly from the examined surface and partially from the subsurface area with an average thickness of 3 nm, which depends on the element as well as the roughness of the area [23] later giving a similar resolution of a later calculated depth profile (Figure 3 and Figure 4). Spectra originating from the elements forming the oxide passivation layer do not change shape or position with the depth, which indicates chemical homogeneity within the layer. A significant evolution occurs, of course, at the substrate–passivity interface.

### 3.2. Potentiodynamic Test

**Figure 5:**
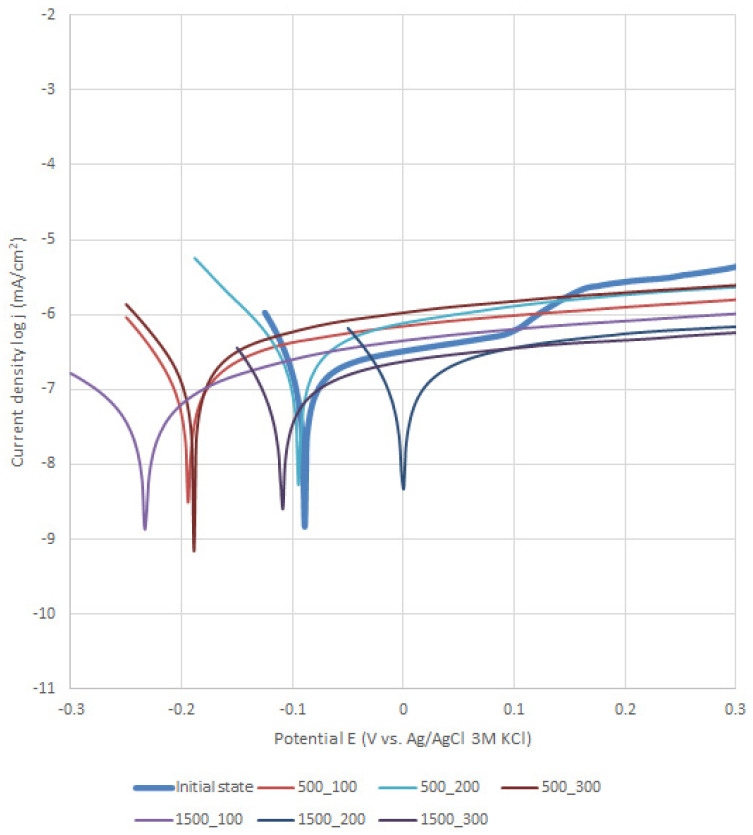
Sandblasting–polarization curves regarding 316LVM with a ZnO layer.

**Figure 6:**
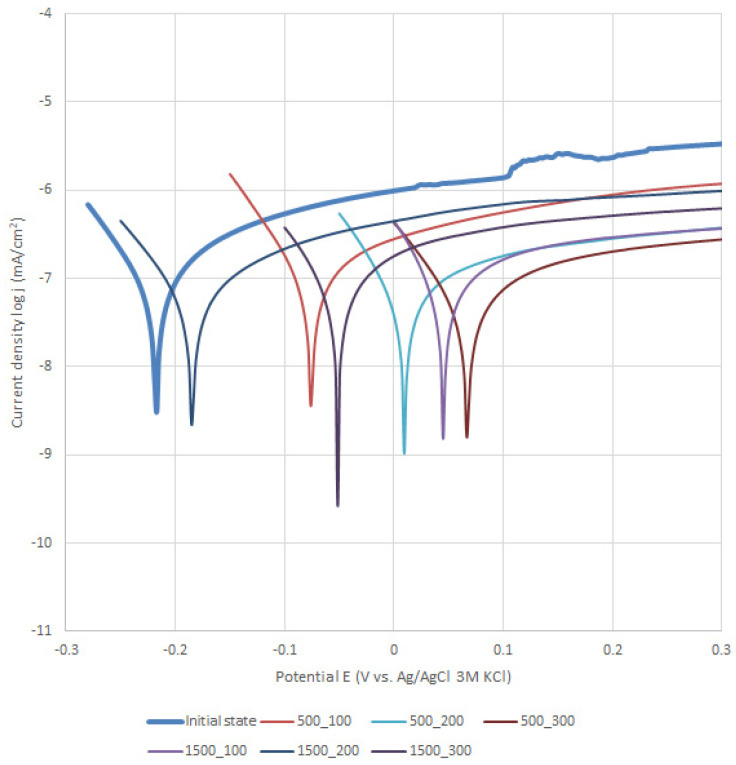
Electropolishing–polarization curves regarding 316LVM with a ZnO layer.

The results of the potentiodynamic tests of pitting corrosion resistance are presented in Table 1 and Figure 5 and Figure 6. On the basis of the obtained curves, it was found that the values of the parameters characterizing the corrosion resistance were different depending on the method of preparing the surface of the metal substrate and the parameters of the deposition of the layer by the ALD method. There was no unequivocal trend in the obtained corrosion parameters in the case of the ZnO layer applied to the steel previously subjected to sandblasting. In particular, depending on the parameters of the ALD process, an increase or decrease in the value of the corrosion potential (Figure 1) and the value of the polarization resistance in comparison to the samples not covered with the ZnO layer (Table 1) were found. For the sandblasted samples with the ZnO layer, an increase in the breakdown potential value was observed. A reduction in the passive current density in the potential range of 0.2–1.0 mV was also found compared to the uncoated samples. On the other hand, for the electropolished samples, and then after the ALD process, it can be clearly stated that the deposited layers had a positive effect on the corrosion resistance of the tested steel. For the samples with the ZnO layer, a decrease in the passive current density in the entire measuring range, an increase in the value of the corrosion potential, an increase in the value of the breakdown potentials and polarization resistance as well as a decrease in the value of the corrosion current were observed (Table 1).

### 3.3. Impedance Test

**Figure 7:**
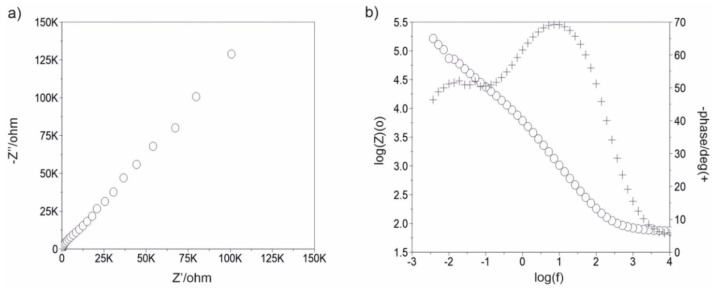
Impedance spectra determined for the initial state of the sandblasted samples: (**a**) Nyquist diagram, (**b**) Bode diagram.

**Figure 8:**
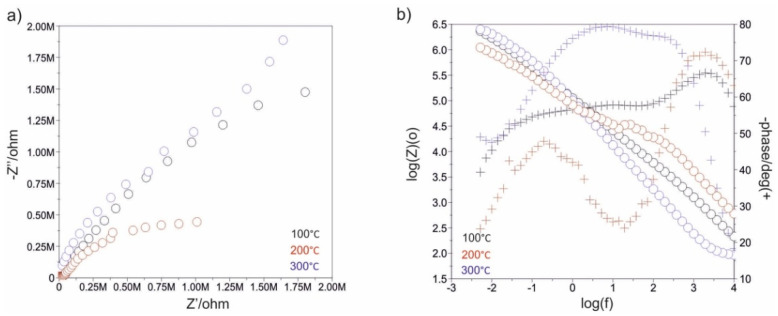
Impedance spectra determined for the ALD process in 500 cycles for the sandblasted samples: (**a**) Nyquist diagram, (**b**) Bode diagram.

**Figure 9:**
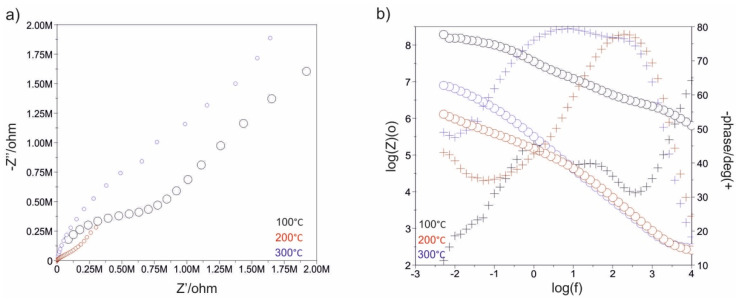
Impedance spectra determined for the ALD process in 1500 cycles for the sandblasted samples: (**a**) Nyquist diagram, (**b**) Bode diagram.

**Figure 10:**
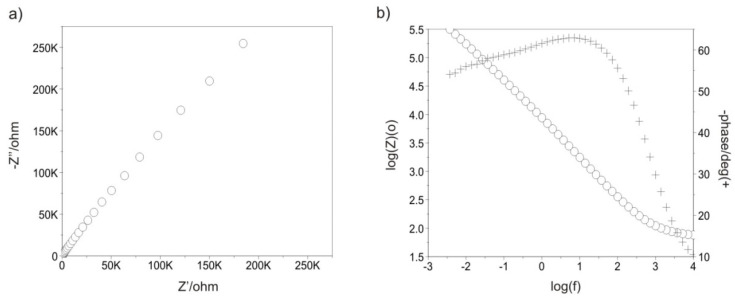
Impedance spectra determined for the initial state of the electropolished samples: (**a**) Nyquist diagram, (**b**) Bode diagram.

**Figure 11:**
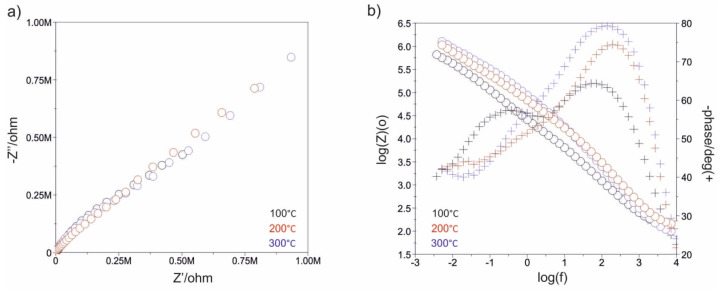
Impedance spectra determined for the ALD process in 500 cycles for the electropolished samples: (**a**) Nyquist diagram, (**b**) Bode diagram.

**Figure 12:**
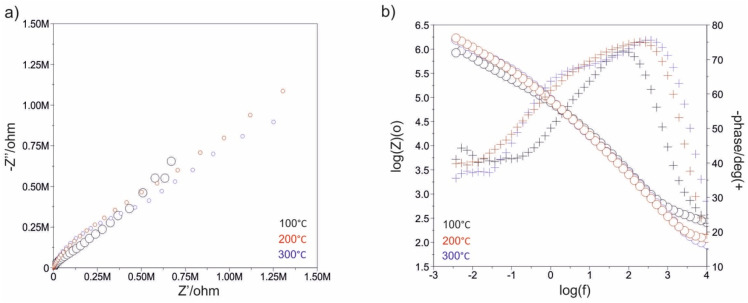
Impedance spectra determined for the ALD process in 1500 cycles for the electropolished samples: (**a**) Nyquist diagram, (**b**) Bode diagram.

Figure 7, Figure 8, Figure 9, Figure 10, Figure 11 and Figure 12 show the impedance spectra recorded during the impedance testing.

**Figure 13:**
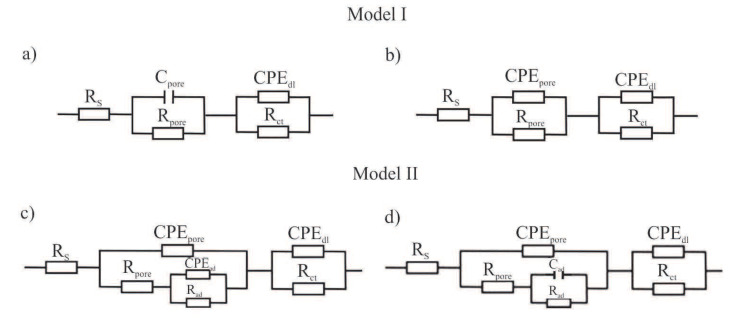
Alternative electrical circuits representing the corrosion systems: (**a**) sandblasted_initial state, (**b**) electropolished and sandblasted—500_300, (**c**) electropolished with ZnO layers, (**d**) sandblasted with ZnO layers besides 500_300.

Characterization of the interface impedance of the electrode–ZnO layer–solution in the process of ZnO layer deposition on the surface of the AISI 316LVM steel by the ALD method was made by approximating the EIS experimental data using physical electrical models of equivalent circuits (Figure 13).

Model I: The passive film and electrical double layer are assumed to show ideal capacitive behavior or non-ideal capacitive behavior. R_pore_ and C_pore_/CPE_pore_ are representatives of the electrical porous layer, whereas R_ct_ and CPE_dl_ represent the resistive and non-ideal capacitive behavior of the passive film (double layer). According to this model, the two R and CPE/C elements are connected in series with the solution resistance (RS) [24]. These models describe the formation of two loops in a Nyquist diagram. A high frequency recording loop, the diameter of which depends on the potential, corresponds to the activity of the oxide film (ZnO). This is determined by the serial resistance R_pore_ and the space charge capacitance C_pore_. However, the low-frequency loop is related to the surface boundary of the oxide layer–solution. A R_ct_ and CPE_dl_ sub-circuit was employed to describe the low-frequency region between 11 and 0.001 Hz.

Model II: In this model, the passive film is considered to have a porous structure and to show non-ideal capacitive behavior. R_pore_ is the electrolyte resistance inside the pores and CPE_pore_ is associated with the oxide film. R_ad_ is the charge transfer resistance of the electrochemical processes taking place inside the pore and C_ad_/CPE_ad_ is associated with an adsorption layer. R_ct_ and CPE_dl_ represent the resistive and non-ideal capacitive behavior of the passive film (double layer) [24]. On the basis of equivalent circuit diagrams, characteristic values describing corrosion resistance were determined for a different number of cycles of the deposition process of the ZnO layer (Table 2). 

### 3.4. Scratch Test

**Figure 14:**
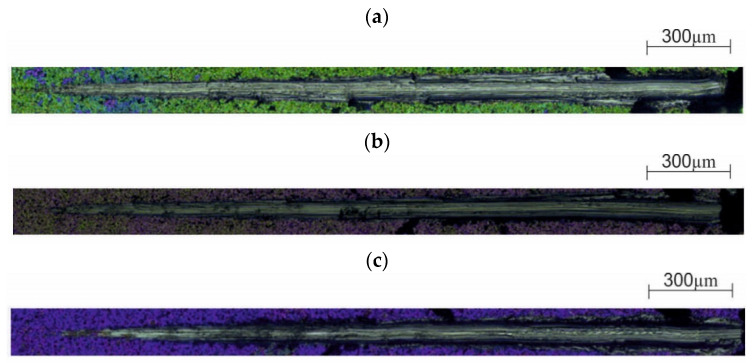
Results of the adhesion tests for the ZnO layer deposited at 1500 cycles (sandblasted): (**a**) 100 °C, (**b**) 200 °C, (**c**) 300 °C.

**Figure 15:**
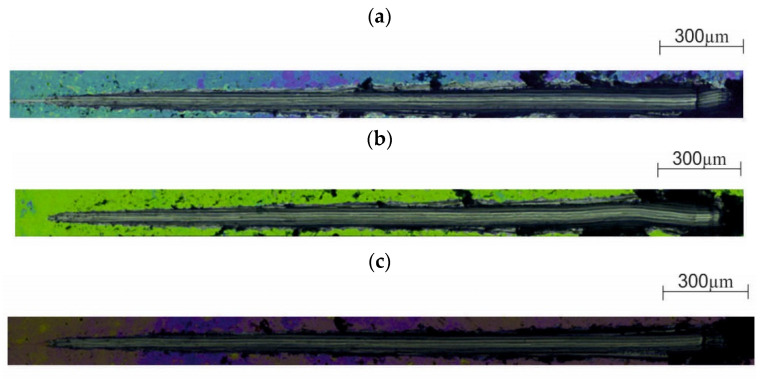
Results of the adhesion tests for the ZnO layer deposited at 1500 cycles (electropolished): (**a**) 100 °C, (**b**) 200 °C, (**c**) 300 °C.

On the basis of the research, different values of adhesion to the substrate were found depending on the method of its preparation (Table 3, Figure 14 and Figure 15). It was observed that the layers deposited on the substrate previously subjected to the sandblasting process showed greater adhesion. Moreover, it was found that with the increase of the temperature of the deposition process, the adhesion of the ZnO layer to the substrate increased, regardless of the method of its preparation. However, no significant differences in the force values were observed depending on the number of cycles of the deposition process.

### 3.5. Scanning Electron Microscope (SEM)

**Figure 16:**
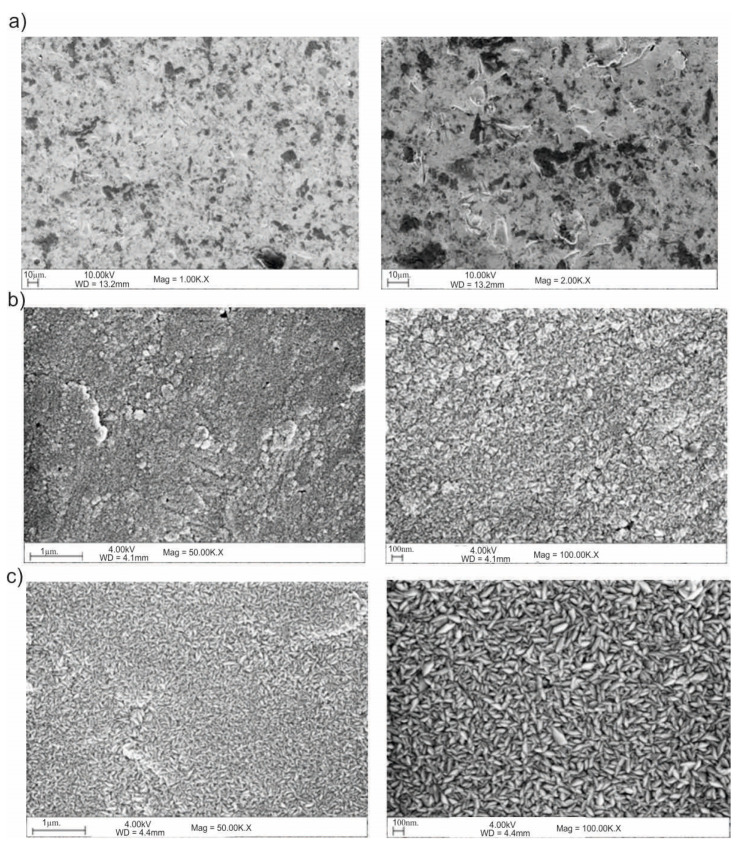
Example of the surface morphology in ZnO-coated samples: (**a**) initial state; (**b**) 500_100°C, (**c**) 1500_100°C.

**Figure 17:**
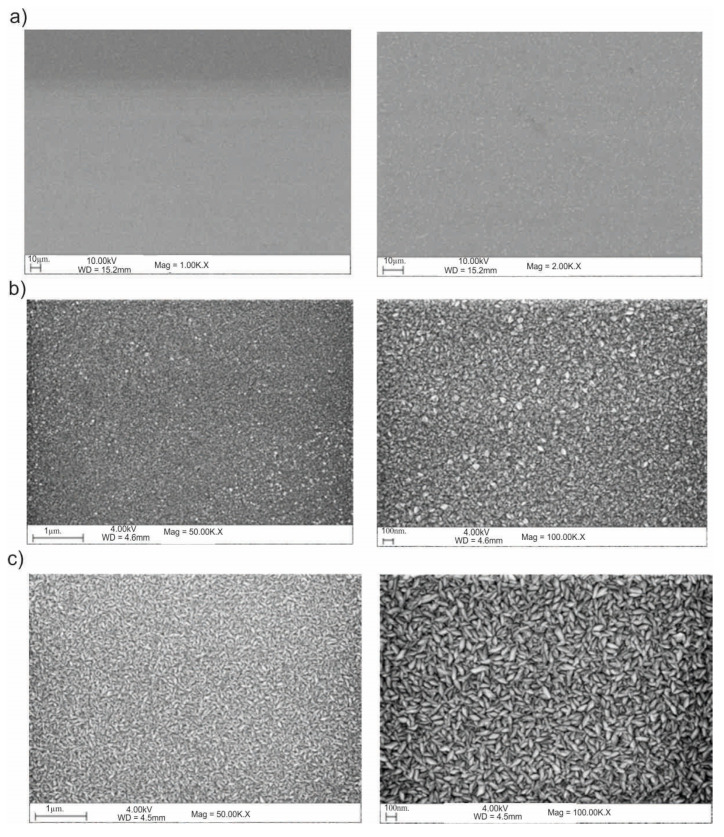
SEM of the sample surface morphology for the electropolished samples: (**a**) initial state, (**b**) 500_200 °C, (**c**) 1500_200 °C.

The results of the SEM observations are shown in Figure 16 and Figure 17. Based on the obtained images, different structural morphologies were found depending on the method of surface preparation.

### 3.6. Wettability

**Figure 18:**
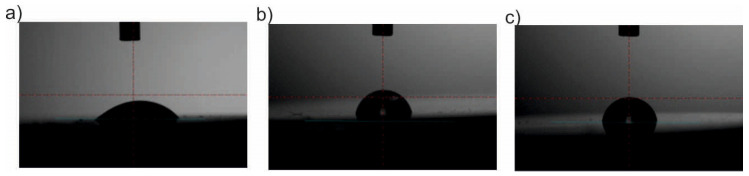
Examples of sessile drops applied to a sandblasted sample: (**a**) initial state; (**b**) 500_200 °C; (**c**) 1500_200 °C.

**Figure 19:**
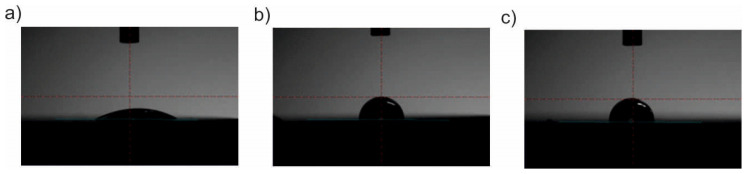
Examples of sessile drops applied to an electropolished sample: (**a**) initial state; (**b**) 500_200 °C; (**c**) 1500_200 °C.

The results of the measurements of the surface wettability are presented in Table 4 and Figure 18 and Figure 19. On the basis of the obtained results, different values of the wetting angle were found depending on the method of surface preparation. There were no significant differences in the values of the angle θ depending on the sandblasting or electropolishing process used. In both cases, the surface was hydrophilic. The deposition of the ZnO layer, regardless of the process parameters used, increased the value of the wetting angle θ, and thus changed the character of the surface from hydrophilic to hydrophobic. Moreover, no significant changes in the angle values were observed for the various parameters of the deposition process (i.e. the number of cycles and temperature).

## 4. Discussion

Modification of the surface of metal biomaterials has a fundamental impact on their physicochemical properties. In addition to the deposition of surface layers in the form of oxides, sulfides, or carbides, it is also important to properly prepare the metallic substrate. This is very important as many surface treatments reflect the surface topography. Hence, they take over some of the physicochemical properties. Therefore, the work proposes the deposition of composite ZnO layers with different process parameters on previously electropolished or sandblasted 316LVM steel substrates. First, the chemical analysis of the deposited ZnO layers was examined on previously electropolished or sandblasted surfaces of 316 LVM steel. On the basis of the conducted tests, no significant differences were found in the distribution of the chemical constitution of the ZnO layer depending on the number of cycles used or the temperature of the process. The spectra originating from the elements forming the oxide passivation layer did not change their shape or position with the depth, which indicates the chemical homogeneity of the ZnO layer. It is evident that a significant change occurred at the substrate–passivation layer interface. The conducted in depth Auger spectroscopic detailed analysis revealed the existence of chromium oxide CrO thin layer of the oxide–steel interface shown in Figure 4. For samples previously polished, slight differences were observed at the interface depending on the temperature. The appearance of a CrO layer in the interphase was found, the thickness of which was greater, the higher the temperature of the process (Figure 4), which was confirmed by the potentiodynamic studies. Based on the obtained results, it was found that in the case of previous electrochemical polishing, regardless of the application parameters, a favorable increase in corrosion resistance was observed (Table 1, Figure 6). In particular, a decrease in the passive current density in the entire measuring range was observed, along with an increase in the value of the corrosion potential, breakdown potential, and the polarization resistance, and a decrease in the value of the corrosion current for the samples with the ZnO layer. The analysis of the values of the determined parameters of corrosion resistance in the case of these samples showed that the samples in the 500-cycle ALD process at the temperatures of 200 °C and 300 °C (Table 1) obtained higher resistance. This is due to the formation of the CrO layer, the thickness of which was greater the higher the process temperature. On the other hand, in the case of the samples previously sandblasted, a slight improvement in corrosion resistance was found (Table 2). This is most likely related to the sandblasting process itself, which bombards the surface with sand at the appropriate pressure. In this case, the beneficial effect of the ZnO layer was only observed after 1500 cycles, regardless of the process temperature. Additionally, the deposited layers were tested using electrochemical impedance spectroscopy (EIS). This method allows for the analysis and interpretation of processes and phenomena occurring at the interface between the implant and the tissue environment. In the presented Nyquist diagrams (Figure 7a, Figure 8a, Figure 9a, Figure 10a, Figure 11a and Figure 12a), near the origin of the coordinates system, variously distorted fragments of semicircles are visible, which in some cases turn into a linear dependence of the imaginary impedance component (Z’’) on the real (Z’). The results of the tests showed that the impedance modulus of the systems, regardless of the type of substrate and the parameters of the deposition of the ZnO layers, decreased with increasing frequency. The deposition process of the layer on the 316LVM steel substrates resulted in high electrochemical stability of the surface layer as evidenced by the relatively high value of the charge transfer resistance R_ct_. The obtained forms of the characteristics for the polished samples with the ZnO layer indicate the presence of a porous oxide layer as a result of the action of the solution. In the case of these samples, a change in the angle of inclination of the rectilinear section was observed in the low-frequency part of the spectrum, going from the angle characteristic for Warburg impedance (45°) to the angle characteristic for membrane-type layers. This is due to the reaction at the layer–solution interface and can have a major influence on the formation of the biofilm. This is probably due to the partial degradation of the ZnO layer in the surface sublayer while producing membrane-type films in the inner layer (adsorption layer). Studies of the adhesion of ZnO layers to the substrate showed an increase in layer adhesion depending on the temperature of the deposition process. It was found that the higher the process temperature, the higher the adhesion of the layer to the substrate. There were no significant differences in the value of the critical force, which is a measure of adhesion, depending on the number of cycles used. The previously sandblasted samples showed better adhesion. This relates to their greater roughness and greater surface development (Figure 16).

In the final stage, tests of surface wettability were carried out, which showed an increase in the contact angle of the ZnO layers, regardless of the parameters of the deposition process, compared to the initial state. It was found that the ZnO layers changed the surface character from hydrophilic to hydrophobic, which is a favorable phenomenon because a higher value of the contact angle, for example, reduces protein adsorption and limits the formation of microorganisms on the implant surface. There were no differences in the values of the contact angle depending on the number of cycles and the deposition temperature.

## 5. Conclusions

To sum up, the ZnO layer deposited on the electropolished substrate had better physicochemical properties compared to the sandblasted substrate. More favorable electrochemical properties were observed including pitting corrosion resistance and higher barrier properties of the ZnO layer deposited on the electropolished steel. This is due to the formation of a CrO layer after the electropolishing process. The electropolished substrate also caused a favorable increase in the contact angle and changed the character of the surface from hydrophilic to hydrophobic. This is an advantageous phenomenon when applying antibacterial layers in order to reduce the adhesion of microorganisms. On the other hand, the research on the adhesion of ZnO layers to the substrate did not show any significant differences in the value of the force of an electropolished or sandblasted substrate. The study showed the effect of temperature and the number of cycles of the deposition process of the obtained properties. The ZnO layer deposited at the temperature of 200–300 °C for 1500 cycles of the deposition process was characterized by the most advantageous physicochemical properties. For samples prepared in this way, research will be carried out with the use of *S. aureus* and *E. coli* reference bacteria.

## Figures and Tables

**Table 1:** Results of the potentiodynamic tests for the surface-modified 316LVM steel.

Number	Temp, °C	Cycles	E_corr_	E_b_	B_a_	B_c_	R_p_	i_corr_
			mV	mV	mV	mV	kΩ⋅cm^2^	µA/cm^2^
**Sandblasted**								
1	Initial state		−87	+1242	+46.7	−28	0.13	55.13
2	100	500	−184	+1462	+48	−37.8	0.15	47.01
3		1500	−167	-	+45.1	−42.9	0.49	14.65
4	200	500	−90	+1481	+49.5	−37	0.07	97.73
5		1500	+3	+1532	+47.8	37.5	0.16	43.73
6	300	500	−132	+1423	+45.2	−40.8	0.10	70.96
7		1500	−96	+1544	+47.2	−35.3	0.27	25.63
**Electropolished**								
1	Initial state		−211	+1239	+43.4	−43.1	0.19	37.33
2	100	500	−71	+1356	+48.5	−37.4	0.18	39.75
3		1500	+49	+1640	+47.5	−35.6	0.23	29.60
4	200	500	+14	+1537	+47.6	−37.7	0.29	24.34
5		1500	−118	+1637	+47.8	−40.5	0.29	24.93
6	300	500	+72	+1523	+45.4	−37.5	0.37	17.95
7		1500	−46	+1683	+44.1	−39.5	0.24	29.37

**Table 2:** Electrochemical Impedance Spectroscopy (EIS) results for the surface-modified 316LVM steel.

Samples	R_ad_, kΩ·cm^2^	C_ad_, µF	CPE_ad_		R_pore_, kΩ·cm^2^	CPE_pore_		R_ct_ ,kΩ·cm^2^	CPE_dl_		E_OCP_, mV
			Y_0_, Ω^−1^cm^−2^s^−n^	n		Y_0_, Ω^−1^cm^−2^s^−n^	n		Y_0_, Ω^−1^cm^−2^s^−n^	n	
Sandblasted *	-	-	-	-	7	-	-	538	0.8050 × 10^−4^	0.72	−71
500_100	1	6	-	-	274	0.1348 × 10^−4^	0.86	1252	0.8123 × 10^−4^	0.71	−79
1500_100	2	28	-	-	2374	0.1578 × 10^−4^	0.88	2852	0.8578 × 10^−4^	0.69	−81
500_200	5	29	-	-	1129	0.1085 × 10^−4^	0.79	1850	0.5144 × 10^−4^	0.68	−93
1500_200	25	8	-	-	1064	0.1686 × 10^−4^	0.91	1138	0.2906 × 10^−4^	0.75	−75
500_300	-	-	-	-	3020	0.3840 × 10^−4^	0.94	7460	0.2382 × 10^−4^	0.71	−18
1500_300	-	-	-	-	3064	0.1756 × 10^−4^	0.93	6838	0.2226 × 10^−4^	0.73	−72
Electropolished	-	-	-	-	21	0.3362 × 10^−4^	0.83	1221	0.4306 × 10^−4^	0.79	−54
500_100	10	-	0.1854 × 10^−4^	0.76	102	0.1219 × 10^−4^	0.69	1210	0.1372 × 10^−4^	0.96	−36
1500_100	16	-	0.1890 × 10^−4^	0.85	120	0.4740 × 10^−4^	0.87	2566	0.1042 × 10^−4^	0.68	+102
500_200	14	-	0.1702 × 10^−4^	0.89	146	0.4922 × 10^−4^	0.84	1904	0.1158 × 10^−4^	0.80	+74
1500_200	26	-	0.3217 × 10^−4^	0.81	355	0.3986 × 10^−4^	0.87	2642	0.1109 × 10^−4^	0.83	+51
500_300	25	-	0.1908 × 10^−4^	0.86	281	0.3704 × 10^−4^	0.89	2201	0.1241 × 10^−4^	0.84	+145
1500_300	5	-	0.2707 × 10^−4^	0.84	410	0.2886 × 10^−4^	0.91	2231	0.1228 × 10^−4^	0.83	+68

Rs = 64 ± 1 Ω∙cm^2^; *: C_pore_ = 47 µF.

**Table 3:** The adhesion of the ZnO layer to the substrate made of 316 LVM steel.

Surface Modification		Failure of the Layer	The Value of Registered Indenter Load Fn, N				
Temp, °C	Cycles		Measurement 1	Measurement 2	Measurement 3	Average	STD
**Sandblasted**							
100	500	Complete break Lc_2_	0.94	0.91	0.99	1.08	±0.04
	1500	Complete break Lc_2_	1.24	1.20	1.22	1.57	±0.02
200	500	Complete break Lc_2_	1.95	1.90	1.96	1.64	±0.03
	1500	Complete break Lc_2_	1.35	1.33	1.35	1.93	±0.01
300	500	Complete break Lc_2_	2.52	2.50	2.55	2.92	±0.02
	1500	Complete break Lc_2_	3.54	3.25	3.20	3.33	±0.18
**Electropolished**							
100	500	Complete break Lc_2_	0.22	0.25	0.19	0.13	±0.03
	1500	Complete break Lc_2_	0.04	0.06	0.06	0.13	±0.01
200	500	Complete break Lc_2_	0.21	0.23	0.20	0.62	±0.01
	1500	Complete break Lc_2_	1.08	1.02	1.01	1.16	±0.03
300	500	Complete break Lc_2_	1.38	1.23	1.28	1.34	±0.07
	1500	Complete break Lc_2_	1.35	1.41	1.44	1.40	±0.04

**Table 4:** Wettability and surface free energy results for the surface-modified 316LVM steel.

Sample	Contact Angle θ_avr_. °		Polar Component γ_s_^p^. mJ/m^2^	Dispersion Component γ_s_^d^. mJ/m^2^	Surface Energy (SEP) γ^s^. mJ/m^2^
	Distilled water	Diiodomethane			
**Sandblasted**					
initial state	47.92 ± 5.68	55.17 ± 0.4	38.17 ± 0.23	12.97 ± 0.11	51.14 ± 0.33
500_100	98.39 ± 1.02	58.42 ± 0.23	0.52 ± 0.30	30.86 ± 0.28	31.39 ± 0.26
1500_100	114.46 ± 2.32	55.89 ± 0.79	1.52 ± 0.09	41.64 ± 0.83	43.16 ± 0.93
500_200	101.60 ± 3.37	57.14 ± 0.25	0.08 ± 0.03	33.5 ± 0.98	33.59 ± 0.82
1500_200	95.60 ± 1.46	57.56 ± 1.02	0.63 ± 0.69	29.23 ± 0.67	30.17 ± 0.59
500_300	100.72 ± 3.13	61.26 ± 0.2	0.36 ± 0.01	29.49 ± 0.18	29.86 ± 0.16
1500_300	93.47 ± 3.65	55.67 ± 2.07	1.4 ± 0.23	30.51 ± 1.7	31.9 ± 1.46
**Electropolished**					
initial state	38.53 ± 4.82	50.97 ± 1.68	45.20 ± 1.02	13.06 ± 0.85	58.26 ± 0.17
500_100	96.52 ± 5.63	55.32 ± 1.42	0.64 ± 0.11	32.38 ± 1.19	33.03 ± 1.08
1500_100	108.24 ± 1.77	56.45 ± 0.25	0.26 ± 0.01	37.68 ± 0.23	37.95 ± 0.23
500_200	100.75 ± 2.93	57.45 ± 0.004	0.16 ± 0.01	32.79 ± 0.23	32.95 ± 0.22
1500_200	101.41 ± 2.46	58.39 ± 3.65	0.16 ± 0.13	32.32 ± 3.16	32.49 ± 3.03
500_300	106.74 ± 2.04	58.96 ± 1.97	0.05 ± 0.03	34.60 ± 1.78	34.65 ± 1.82
1500_300	102.60 ± 3.05	55.01 ± 1.2	0.005 ± 0.007	36.13 ± 1.41	36.14 ± 1.40
